# Sorafenib-Induced Hand-Foot Skin Reaction in a Patient With Acute Myeloid Leukemia Following Haploidentical Stem Cell Transplantation: A Case Report

**DOI:** 10.7759/cureus.116921

**Published:** 2026-09-25

**Authors:** Christa Davis, Arun Vignesh, Sudha Rangarajan, Murugan Sundaram, Adikrishnan Swaminathan

**Affiliations:** 1 Dermatology, Venereology and Leprosy, Sri Ramachandra Institute of Higher Education and Research, Chennai, IND

**Keywords:** acute myeloid leukemia, adverse drug reaction, flt3 mutation, hand-foot skin reaction, haploidentical stem cell transplantation, multikinase inhibitor, sorafenib, tyrosine kinase inhibitor

## Abstract

Sorafenib is an oral multikinase inhibitor approved for advanced hepatocellular carcinoma, renal cell carcinoma, and maintenance therapy in selected patients with FMS-like tyrosine kinase (FLT3)-mutated acute myeloid leukemia following allogeneic hematopoietic stem cell transplantation.

We report the case of a 35-year-old woman with FLT3 internal tandem duplication (FLT3-ITD) and nucleophosmin 1 (NPM1)-positive acute myeloid leukemia (AML) who developed painful hyperkeratotic plantar plaques approximately eight weeks after initiating sorafenib therapy. Clinical examination revealed multiple symmetrical hyperkeratotic plaques involving the pressure-bearing areas of both soles, associated with pain and tenderness, resulting in significant impairment of daily activities. She was diagnosed with sorafenib-induced hand-foot skin reaction (HFSR) and was managed with topical keratolytic and corticosteroid therapy.

This case highlights the importance of early recognition and prompt management of sorafenib-induced HFSRs to minimize morbidity and maintain oncological treatment.

## Introduction

Sorafenib is an oral multikinase inhibitor that targets RAF kinases, vascular endothelial growth factor receptors (VEGFR), platelet-derived growth factor receptors (PDGFR), and other intracellular signaling pathways, thereby inhibiting tumor proliferation and angiogenesis. It has demonstrated efficacy in hepatocellular carcinoma, renal cell carcinoma, and FLT3-mutated acute myeloid leukemia (AML), particularly in the post-transplant setting [1,2].

Cutaneous adverse events are common during sorafenib therapy, with hand-foot skin reactions (HFSRs) being one of the most characteristic toxicities [3]. Unlike conventional chemotherapy-induced hand-foot syndrome, HFSR predominantly affects pressure-bearing and friction-prone areas, presenting with painful hyperkeratotic plaques, erythema, blistering, and dysesthesia [4].

HFSR typically develops within the first 2-3 weeks of sorafenib therapy; therefore, the relatively delayed onset in our patient highlights the clinical importance of recognizing HFSR beyond the usual timeframe. We report a case of sorafenib-induced HFSR in a patient with FMS-like tyrosine kinase 3 (FLT3)-mutated AML following haploidentical hematopoietic stem cell transplantation.

## Case presentation

A 35-year-old woman diagnosed with AML harboring FLT3-ITD and NPM1 mutations underwent haploidentical hematopoietic stem cell transplantation (haplo-HSCT). Sorafenib was initiated at a dose of 200 mg twice daily as post-transplant maintenance therapy. After two months, the dose was increased to 400 mg twice daily.

Approximately eight weeks after initiating sorafenib therapy, the patient initially developed a burning sensation involving both soles. Over the following days, multiple painful hyperkeratotic lesions appeared over the pressure-bearing areas of the feet, gradually increasing in size and number, and interfering with routine activities.

Dermatological examination revealed multiple well-defined symmetrical, hyperkeratotic plaques involving the heels, lateral plantar surfaces, first metatarsal heads, and great toes bilaterally. The orange-brown discoloration visible in the clinical images is due to recent henna (mehendi) application and is unrelated to the cutaneous manifestations of sorafenib-induced HFSR (Figure 1). 

**Figure 1:**
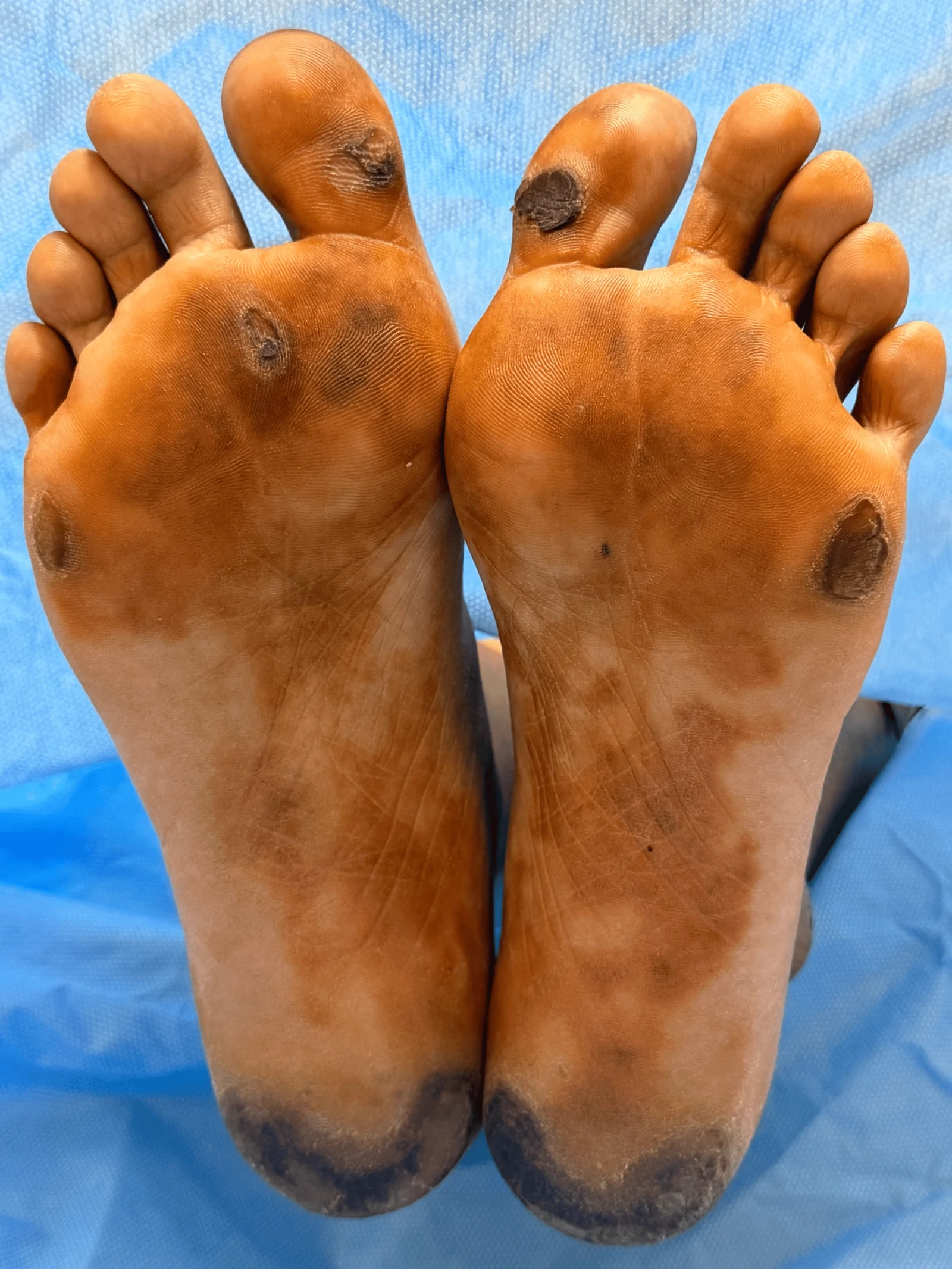
Bilateral plantar surfaces demonstrating multiple symmetrical hyperkeratotic plaques over pressure-bearing areas, including the great toes, metatarsal heads, lateral plantar surfaces, and heels.

Marked hyperpigmentation and hyperkeratosis of the heels were observed (Figure 2).

**Figure 2:**
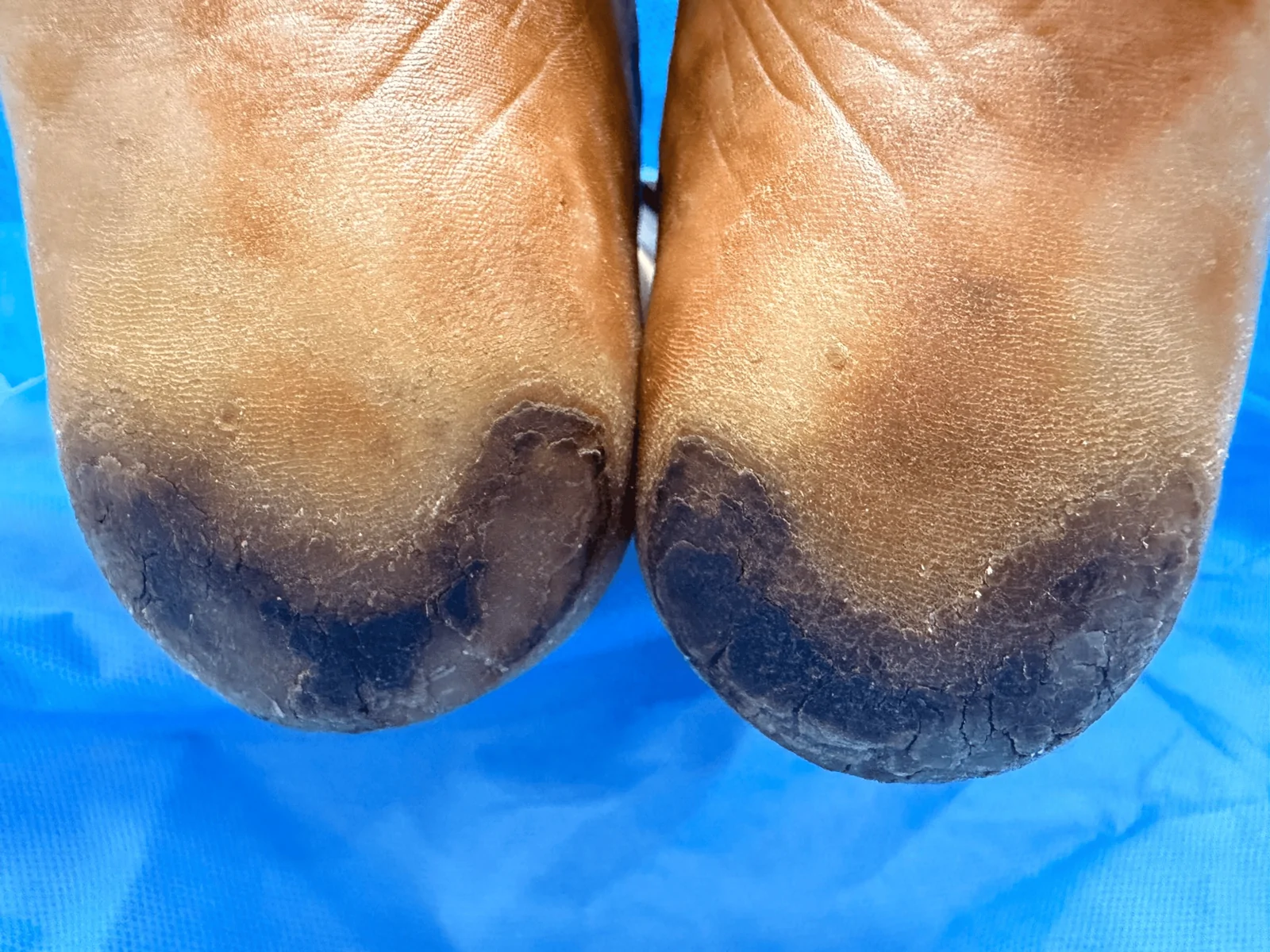
Prominent hyperkeratosis and blackish-brown hyperpigmentation involving both heels, representing sorafenib-induced plantar toxicity.

Based on the characteristic morphology, distribution, and temporal relationship with sorafenib therapy, a clinical diagnosis of HFSR was established.

The patient was treated with a glycolic acid- and urea-containing keratolytic and emollient cream twice daily, along with clobetasol propionate 0.05% and salicylic acid 6% ointment once nightly, in addition to counseling regarding friction reduction, pressure off-loading, and skin care measures.

After two weeks of treatment, the patient demonstrated marked clinical improvement with a substantial reduction in pain and inflammation. Examination revealed regression of the plaques with mild residual plantar hyperkeratosis (Figures 3, 4). Sorafenib was neither discontinued nor dose-modified, given the clinical improvement with topical treatment and supportive measures, and was therefore continued.

**Figure 3:**
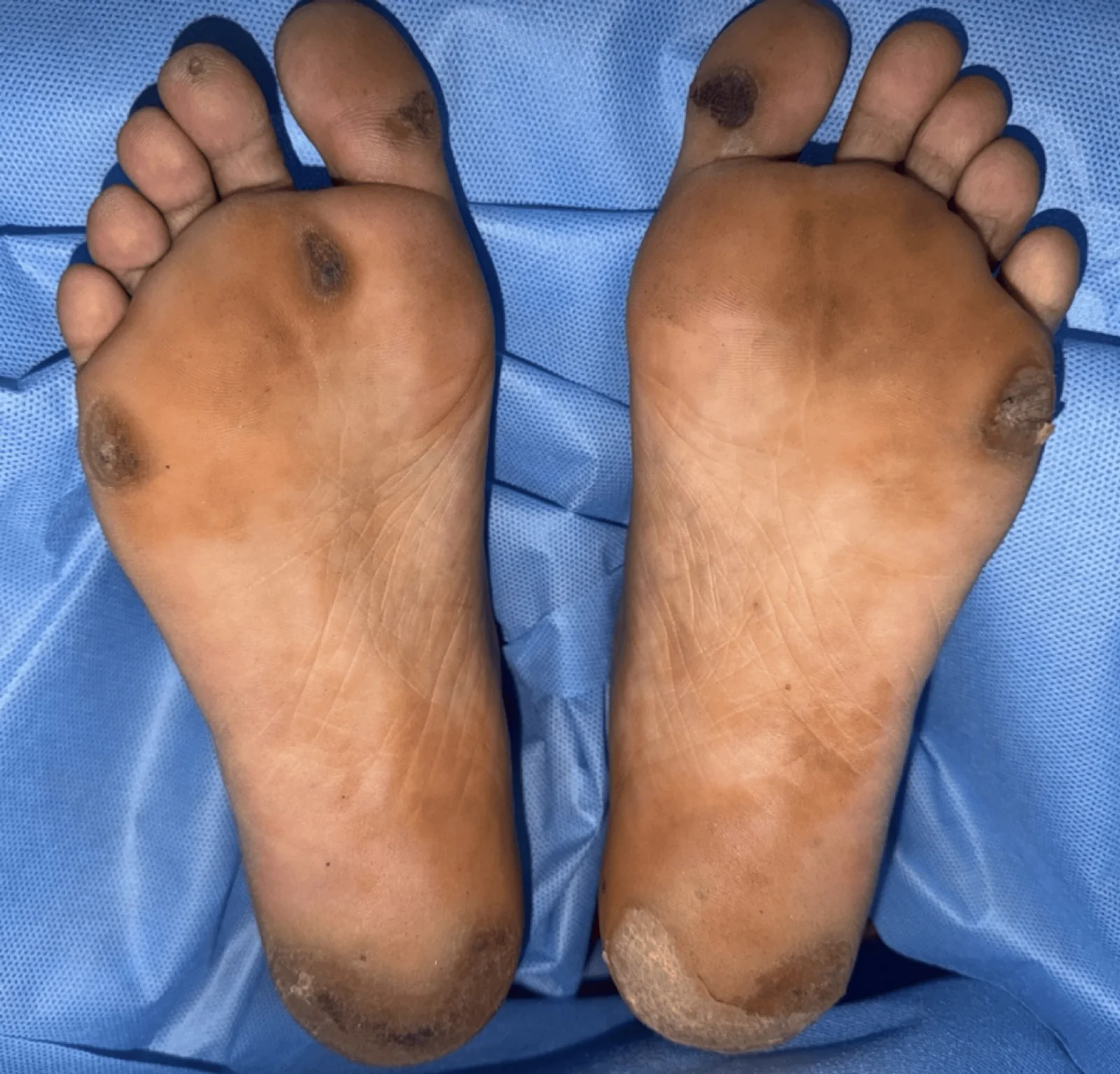
Follow-up photograph of the bilateral plantar surfaces after two weeks of treatment demonstrating marked regression of the hyperkeratotic plaques, with residual post-inflammatory hyperpigmentation over the pressure-bearing areas of the heels, first metatarsal heads, and great toes.

**Figure 4:**
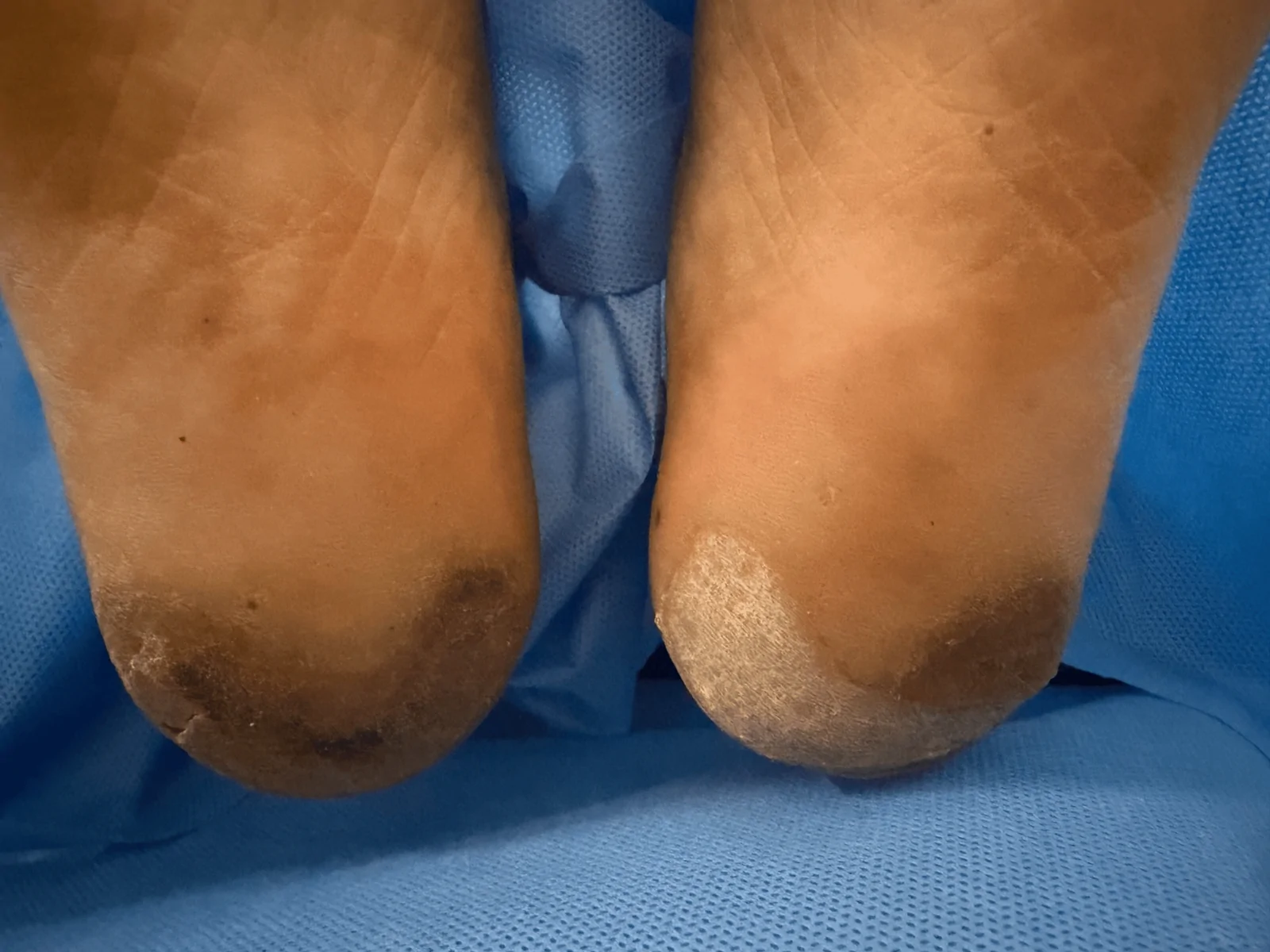
Follow-up photograph of the bilateral heels after two weeks of treatment demonstrating marked improvement in plantar hand-foot skin reaction, with residual mild hyperkeratosis and post-inflammatory hyperpigmentation over the heels.

No new lesions were observed, and the patient reported substantial relief of symptoms.

## Discussion

Sorafenib-induced HFSR results from inhibition of RAF kinases (c-RAF, B-RAF), VEGFR-1/2/3, PDGFR, c-KIT, and FLT3, thereby exerting both anti-proliferative and anti-angiogenic effects. Reported incidences of sorafenib-induced HFSR vary considerably across clinical trials depending on tumor type, treatment regimen, and grading criteria. The reaction typically develops within the first 2-3 weeks of therapy and presents as painful erythematous lesions with dysesthesia, hyperkeratosis, callosity, and occasional blistering [5,6]. Unlike conventional chemotherapy-induced hand-foot syndrome, sorafenib-induced HFSR is dose-dependent and predominantly affects pressure-bearing areas, whereas chemotherapy-induced HFS presents with diffuse palmoplantar erythema, swelling, and dysesthesia, commonly associated with capecitabine, 5-fluorouracil, cytarabine, liposomal doxorubicin, and methotrexate [4]. The salient differences between conventional chemotherapy-induced HFS and sorafenib-induced HFSR are outlined in Table 1.

**Table 1:** Clinical and morphological differences between cytotoxic chemotherapy-induced hand-foot syndrome and sorafenib-induced hand-foot skin reaction. Source: [7,8].

Feature	Hand-foot syndrome (HFS)	Sorafenib-induced hand-foot skin reaction (HFSR)
Associated drugs	Cytotoxic chemotherapeutic agents (e.g., capecitabine, 5-fluorouracil)	Multikinase inhibitors, especially sorafenib
Onset	Days to months after therapy initiation	Typically within 2-3 weeks
Distribution	Diffuse involvement of palms and soles	Localized to pressure/friction-bearing areas
Clinical features	Erythema, edema, pain and desquamation	Painful hyperkeratotic plaques, calluses, blisters with erythema
Hyperkeratosis	Less characteristic	Characteristic; yellowish, painful hyperkeratotic plaques localized to pressure areas, particularly heels and metatarsals

The differential diagnosis of HFSR includes conventional hand-foot syndrome, plantar psoriasis, and contact dermatitis. In this case, the characteristic distribution, morphology, and temporal association with sorafenib therapy favored HFSR.

The characteristic localization of sorafenib-induced HFSR to pressure-bearing areas is thought to result from impaired microvascular repair, with repetitive friction and pressure further contributing to localized toxicity and lesion development [9]. Oral multikinase inhibitors, including sunitinib, regorafenib, axitinib, cabozantinib, and pazopanib, are among the few other agents known to be associated with HFSR [10].

The severity of sorafenib-induced HFSR is graded according to the National Cancer Institute Common Terminology Criteria for Adverse Events (NCI-CTCAE), as outlined in Table 2.

**Table 2:** Comparison of NCI Common Terminology Criteria for Adverse Events (CTCAE) Version 5.0 Hand-Foot Skin Reaction Grading and sorafenib-associated skin toxicity. Source: [4,11].

Grade	NCI-CTCAE Version 5.0 Hand-Foot Skin Reaction (HFSR)	Sorafenib-associated skin toxicity
Grade 1	Mild cutaneous manifestations, including erythema, edema, or hyperkeratosis, without pain or limitation of daily activities	Numbness, dysesthesia, paresthesia, tingling, painless swelling, erythema, or discomfort of the hands and/or feet that do not interfere with activities of daily living (ADL)
Grade 2	Painful cutaneous changes, including peeling, blistering, bleeding, edema, or hyperkeratosis, resulting in limitation of instrumental activities of daily living (IADL)	Painful erythema and swelling of the hands and/or feet that interfere with activities of daily living, often accompanied by hyperkeratosis
Grade 3	Severe painful cutaneous changes, including peeling, blistering, bleeding, edema, or hyperkeratosis, resulting in limitation of self-care activities of daily living (ADL)	Severe blistering, ulceration, moist desquamation, or intense pain causing marked functional impairment and inability to perform activities of daily living

Histopathological findings in HFSR are nonspecific and therefore not essential for diagnosis. The diagnosis is largely clinical, relying on the temporal association with sorafenib therapy and the characteristic distribution of lesions. Reported histological features include parakeratosis, dyskeratosis, and band-like areas of necrotic keratinocytes with superficial bullous lesions [12].

The management of hand-foot skin reactions is guided by the severity of clinical manifestations, with treatment ranging from supportive care to temporary interruption of the offending agent (Table 3).

**Table 3:** Grading, management, and dose modification of sorafenib-induced hand-foot skin reaction. Source: [4].

Severity grade	Clinical features	Management strategies	Dose modification
Grade 1	Asymptomatic cutaneous changes including erythema, edema, or hyperkeratosis without associated discomfort	Avoid exposure to hot water; apply emollient creams regularly; wear padded cotton gloves and socks; topical urea (20%-40%)	Continue current sorafenib dose without modification
Grade 2	Painful cutaneous manifestations, including desquamation, blistering, hemorrhage, edema, or marked hyperkeratosis, resulting in limitation of instrumental activities of daily living	All grade 1 measures; add topical clobetasol propionate 0.05% ointment; topical 2% lidocaine for local analgesia; systemic pain relief with codeine or pregabalin; topical calcipotriol	Reduce dose by 50% for 7-28 days
Grade 3	Severe painful cutaneous changes (desquamation, bullae, hemorrhage, edema, hyperkeratosis); restricts basic self-care activities of daily living	All grade 1 and grade 2 measures as above	Temporarily discontinue sorafenib for a minimum of 7 days and resume after the toxicity improves to grade 0 or 1

Emerging evidence suggests that nicotinamide may have a role in the management of HFSR. In a pilot series involving 10 patients, clinical improvement was attributed to modulation of soluble heparin-binding epidermal growth factor-like growth factor (s-HBEGF)-mediated keratinocyte-endothelial crosstalk, a pathway thought to contribute to the pathogenesis of HFSR. Although these preliminary findings are promising, larger prospective studies are needed to confirm its efficacy [13].

## Conclusions

Sorafenib-induced HFSRs are common yet potentially debilitating adverse effects that may significantly impair the quality of life. Early diagnosis, based on characteristic clinical morphology and distribution, is essential for timely intervention. This case highlights the occurrence of grade 2 HFSR in a patient receiving sorafenib maintenance therapy following haploidentical stem cell transplantation for FLT3-mutated AML. Prompt institution of topical therapy and supportive measures is crucial for symptom control and continuation of oncological treatment, thereby reducing morbidity.
